# Supplementary material for: Pharmacological Inhibition of NHE1 Protein Increases White Matter Resilience and Neurofunctional Recovery after Ischemic Stroke
Source: Int J Mol Sci. 2023 Aug 27;24(17):13289. doi: 10.3390/ijms241713289 (PMC10488118; doi:10.3390/ijms241713289)
Supplement: Supplementary file 1 [file ijms-24-13289-s001.zip › ijms-2582352-supplementary.pdf]

Supplemental Materials

Transient focal ischemia model

Focal cerebral ischemia was induced by occlusion of the left middle cerebral artery (MCA) as described before [1]. Briefly, mice were kept under 1.5% isoflurane anesthesia during the procedure and the core temperature (37.0 °C) was maintained by a small animal temperature controller pad throughout all procedures. After midline skin incision, the left common carotid artery was exposed and the superior thyroid artery and occipital artery branches of the external carotid artery were isolated and coagulated. The animals were subjected to MCA occlusion (MCAO) by introduction of a silicone-coated suture (6-0 monofilament nylon, Doccol, USA) inserted via the external carotid artery. Reperfusion was established by withdrawal of the filament after 60 min of transient MCAO (tMCAO). The incision was closed and the mice were allowed to recover under a heating lamp to maintain the core temperature (36~37 °C) during a 30-60 min recovery period. Sham controls underwent identical procedures without the use of suture. All animals were returned to their cages with free access to food and water after the procedures.

Flow cytometry

Mice were deeply anesthetized with 5% isoflurane and transcardially perfused with ice-cold saline, as described before [1]. After removal of cerebellum and meninges, contralateral (CL) and ipsilateral (IL) hemispheric tissues were separated and cut into small pieces before dissociating into single cell suspensions by gentle physical disruption and enzymatic digestion using a tissue dissociation kit, according to manufacturer's instructions (Miltenyi Biotech Inc., Germany). The single cell suspension sample was centrifuged through a 30/70 Percoll (GE Healthcare, USA) gradient solution at 500×g for 30 min to remove myelin. Brain cells were then collected from the middle layer and washed twice with HBSS containing 1% fetal bovine serum (FBS), before incubating with BUV395-conjugated CD11b (1:400, BD Biosciences), FITC-conjugated CD206 (1:400,BioLegend), eFluor 450-conjugated CD16/32 (1:400, eBioscience), BV605-conjugated CD68 (1:400 BioLegend), Alexa Fluor 700-conjugated CD86 (1:400, BD Biosciences), PE-conjugated Ym1 (1:400, Abcam), and PerCP-Cy5.5-conjugated CD45 (1:400, BioLegend) antibodies for 20 min at 4°C in the dark. At least 10,000 events were recorded from each hemispheric sample using an LSR Fortessa flow cytometer (BD Biosciences, USA) running FACS Diva software (BD Bioseciences, USA) with the following settings: Forward scatter (FSC) V =

275, mode = Lin; Side scatter (SSC) V = 325, mode = Lin; BUV395 V = 490, mode = Log; FITC V = 415, mode = Log; PE V = 500, mode = Log; PerCP-Cy5.5 V = 600, mode = Log; eFluor 450 V = 410, mode = Log; Alexa 700 V = 475, mode = Log; BV605 V = 475, mode = Log. Data was analyzed with FlowJo software (BD Biosciences, USA).

Immunofluorescent staining

Mice were euthanized with overdose of CO_2 and transcardially perfused with 0.1 M PBS (pH 7.4), followed by ice-cold 4 % PFA in 0.1 M PBS as described before [2]. Brains were cryoprotected with 30% sucrose after an overnight post-fixation in 4% PFA [1]. Coronal sections (25 μm thickness) were sectioned using a Leica SM2010R microtome (Leica, Germany) for immunofluorescent staining. The sections were incubated with blocking solution (10% normal goat serum and 0.3% Triton X-100 in PBS) for 1 h at room temperature and were then incubated with mouse monoclonal anti-GFAP (1:200, Cell Signaling Technology, USA) and rabbit polyclonal anti-IBA1 (1:200, FUJIFILM Wako Pure Chemical, Japan); mouse monoclonal anti-Olig2 (1:200, Millipore, USA) and rabbit polyclonal anti-NG2 (1:200, Millipore, USA) or rabbit polyclonal anti-Ki67 (1:200, Millipore, USA) or rabbit polyclonal anti-caspase3 (1:200, Cell Signaling Technology, USA); mouse monoclonal anti-APC (1:200, Millipore, USA) and rabbit polyclonal anti-MBP (1:200, Abcam, USA) antibodies in the blocking solution for overnight at 4°C. After washing in TBS-Triton X-100 (0.3%) for 3×10 min, the sections were incubated with goat anti-mouse Alexa 546-conjuagated IgG (1:200, Thermo Fisher Scientific) and goat anti-rabbit Alexa 488-conjugated IgG (1:200, Thermo Fisher Scientific), or goat anti-mouse Alexa 488-conjuagated IgG (1:200, Invitrogen) and goat anti-rabbit Alexa 546-conjugated IgG (1:200, Invitrogen) in the blocking solution for 1 h. For negative controls, brain sections were stained with the secondary antibodies only. After washing for 3 times, nuclei were stained with DAPI (1:500, Thermo Fisher Scientific) for 15 min at 37°C. Sections were mounted with Vectashield mounting medium (Vector Laboratories). Fluorescent images were captured under 40× objective lens using a Nikon A1R confocal microscope (Olympus, Japan). Identical digital imaging acquisition parameters were used and images were obtained and analyzed in a blinded manner throughout the study.

2,3,5-triphenyltetrazolium chloride (TTC) staining

Mice were euthanized with 5% isoflurane vaporized in N_2O and O_2 (3:2) and decapitated, as described before [1]. The brains were dissected and cut into 4 coronal slices of 2 mm thickness before staining with 2% 2,3,5-triphenyltetrazolium chloride (TTC, Sigma, USA) at 37 °C for 15 min. Infarct volume was calculated using Image J (NIH, USA) with correction for edema as described by Swanson [3]. Briefly, the ischemic area for each slice was calculated by subtracting the non-infarct area in the IL hemisphere from the total area of the CL hemisphere. For swelling assessment, percentage hemispheric swelling (%HSw) is determined by the difference in volume between two hemispheres and then divided by the CL hemispheric volume according to a formula: %HSw = (IL volume – CL volume) / CL volume x 100, as described before [1].

Neurological function tests

Neurological functional deficits in mice were screened in a blinded manner with neurological scoring. Sensorimotor functions were measured with rotarod accelerating test, foot-fault test, adhesive contact test, and adhesive removal test, and cognitive functions were determined with open field test and novel object recognition test, all considered reliable for identifying and quantifying neurological functional deficits in rodent models.

a) Neurological scoring. A neurological deficit grading system [1] was used to evaluate neurological deficit at 1 h reperfusion (0 d), and at 1, 2, 3, 5, 7, 10 and 14 d post-tMCAO. The scores are as follows: 0: no neurological deficit; 1: forelimb flexion when suspended by the tail or failure to extend forepaw fully; 2: shoulder adduction when suspended by the tail; 3: reduced resistance to lateral push; 4: spontaneous movement in all directions with unilateral circling exhibited only if pulled by tail; 5: spontaneous unilateral circling; 6: walk only when stimulated; 7: no response to stimulation; 8: stroke-related death.

b) Rotarod accelerating test. The rotarod test was used to assess motor coordination and balance alterations, as described before [1]. Animals were tested prior to tMCAO and at 2, 3, 5, 7, 10 and 14 d post-tMCAO. The apparatus consisted of a black striated rod separated in four compartments (Model 755; IITC Life Science Inc., USA). Animals were first habituated to a stationary rod for 2 min and then placed on a rotating drum accelerating from 4 to 40 r/min over a 5-min period. The time the animal stayed on the drum was recorded. Three trials were performed with 15-min interval resting periods. The time at which the animal fell off the drum was recorded. Pre-operative training was carried twice per day for consecutive three days.

c) Foot fault test. The foot-fault test is sensitive to deficits in descending motor control, as described previously [4]. Each mouse was placed on a stainless-steel grid floor (20 cm × 40 cm with a mesh size of 4 cm^2) elevated 1 m above the floor. The animals were first habituated to the grid floor for 1 min, and then tested for three trials. Each trial ended with at least 50 steps were made by the forelimb contralateral to the injury hemisphere or after 2 min had elapsed. Data were expressed as a percentage of the foot fault errors in total steps. Pre-operative training was carried twice per day for consecutive three days.

d) Adhesive contact and removal tests. The adhesive contact test and the adhesive removal test were used to measure somatosensory deficits, as described previously [1]. Two pieces of adhesive tape (4 mm × 3 mm) were attached to both forepaws in an alternating sequence and with equal pressure by the experimenter before each trial. The contact time was defined as the time at which the animal first made contact with the tape, and the removal time was defined as the time at which the animal removed the tape. The trial ended after the adhesive patch was removed or after 2 min had elapsed. Pre-operative training was carried twice per day for consecutive three days.

e) Open field test. The open field test was used to detect gross locomotor activity and anxiety-like emotional behavior, as described previously [2]. Each mouse was placed in the center of an open field chamber (50 cm × 50 cm × 50 cm) and monitored for 10 minutes with an overhead video tracking system. Each animal's travelled distance, velocity, and time spent in pre-defined zones (center, corners, or peripheral areas) were recorded.

f) Y-maze spontaneous alternation test. The Y-maze spontaneous alternation test was used to assess spatial working memory, as described previously [2]. Each animal was placed into one arm of the Y-maze and monitored over an 8-min duration with an overhead video tracking system. A sequential list of arms entries was analyzed using an automated Sequence Analysis Tool macro in excel. Spontaneous alternation was only counted when a mouse entered three different arms consecutively. The spontaneous alternation % was calculated as the percentage of the number of triad spontaneous alternation in total arm entries minus two.

g) Novel spatial recognition test. The novel spatial recognition test was used to examine spatial reference memory as described previously [5]. The Y-maze is a y-shaped arena that consists of three arms with visual cues of different shapes and colors on each arm. Each mouse was placed into the starting arm and allowed to explore the Y-maze with one arm blocked for 10 min for habituation. The animals were returned to their home cage for 5 min, then re-introduced into the

maze with all arms open for another 5 min. Each animal's location, path and time were recorded by an overhead video tracking system, and total entries and the time spent (T) in each arm were analyzed. The differentiation index (DI) was calculated as: (T_{novel} – $T_{familiar}$) / T_{total}, and the recognition index (RI) was calculated as: T_{novel} / T_{total}.

Data analysis

Unbiased study design and analyses were used in all the experiments. Blinding of investigators to experimental groups were maintained until data were fully analyzed whenever possible. Data were expressed as mean ± SD or SEM and all data were tested for normal distribution. Not normally distributed data were analyzed by Mann-Whitney U test or other appropriate alternative tests according to the data (GraphPad Prism, USA). Two-tailed Student's *t*-test with 95% confidence was used when comparing two conditions. For more than two conditions, one-way or two-way ANOVA analysis was used, depending on the data. A p value < 0.05 was considered statistically significant. All data were included unless appropriate outlier analysis suggested otherwise.

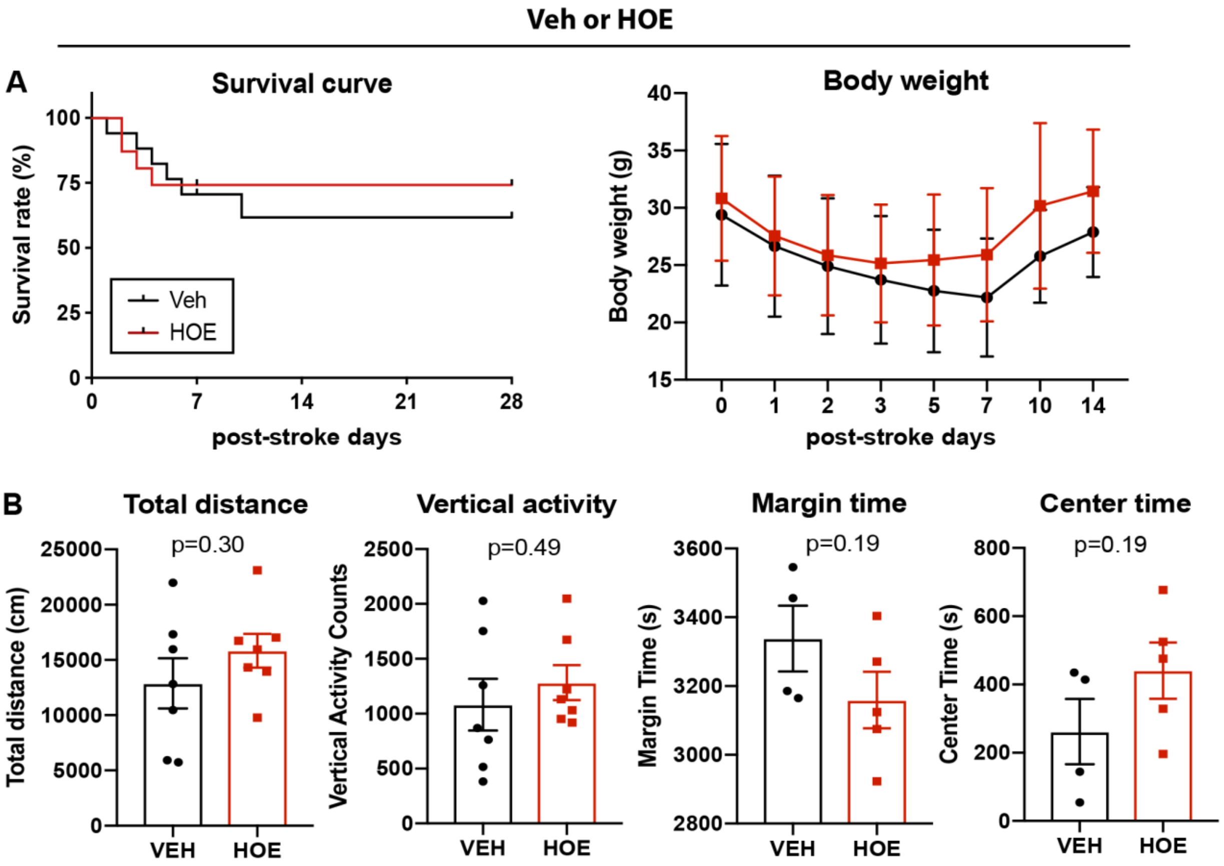

Figure S1. Administration of NHE1 inhibitor HOE642 at 24 h post-stroke did not significantly improve survival rate or anxiety by 28 d in C57/BL6J mice.

A. Survival curve and body weight changes in C57/BL6J mice treated with Veh or HOE642 at 24 h post-stroke. **B.** Open field test at 28 d post-stroke in the same cohort of mice in **Figure 1**. N = 4-7. Data are mean ± SEM.

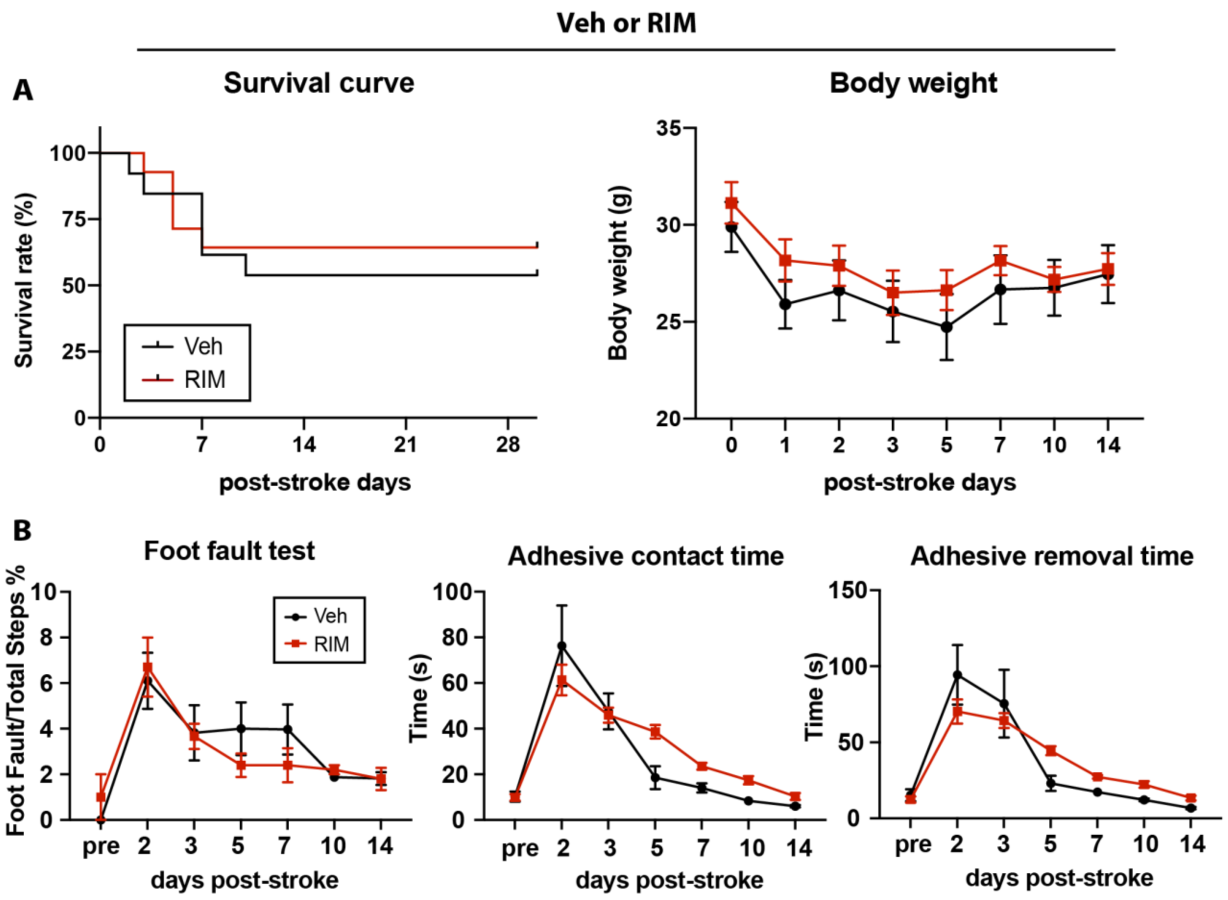

Figure S2. Administration of novel NHE1 inhibitor Rimeporide at 24 h post-stroke showed minimal effects on survival rate by 28 d in C57/BL6J mice.

A. Survival curve and body weight changes in C57/BL6J mice treated with Veh or Rimeporide at 24 h post-stroke. **B.** Foot fault test and adhesive contact/removal tests at 28 d post-stroke in the same cohort of mice in **Figure 4**. N = 5-7. Data are mean ± SEM.

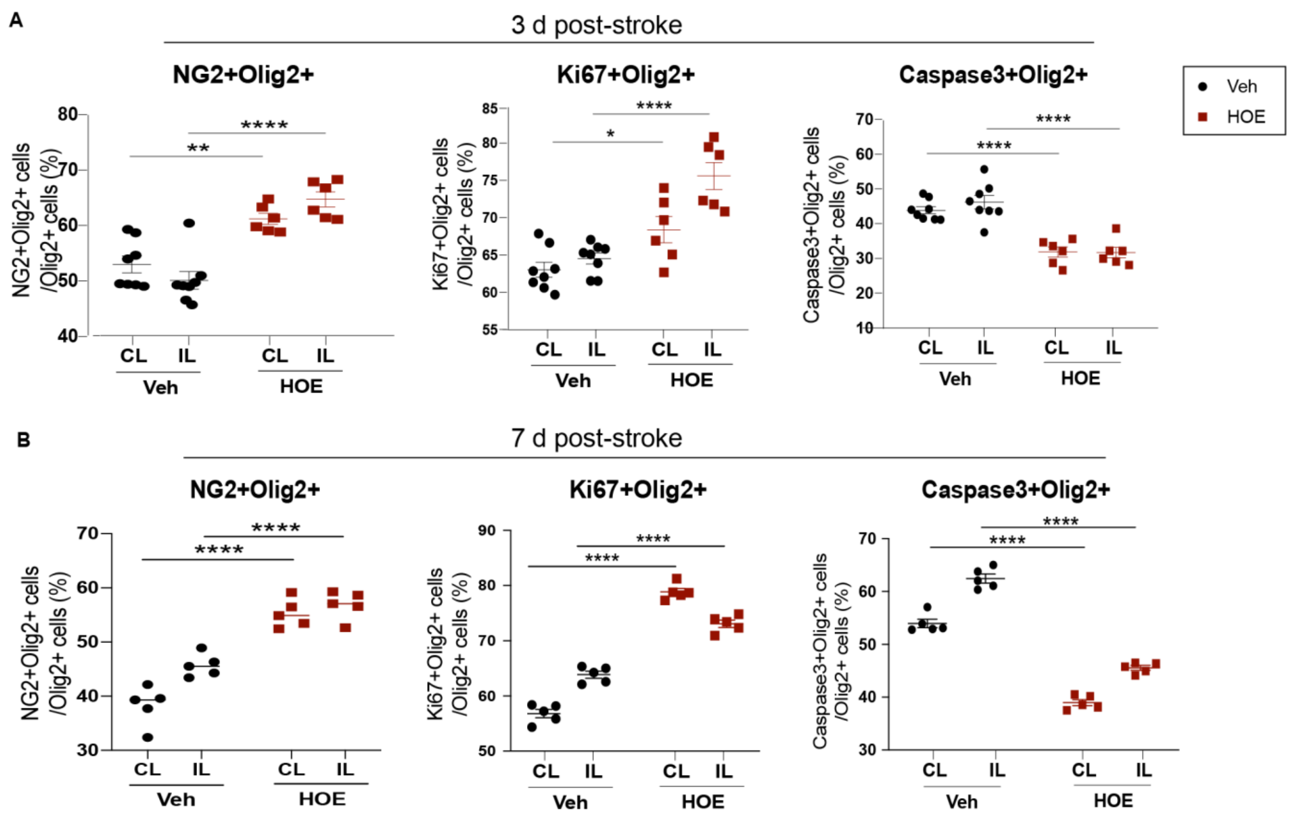

Figure S3. Post-stroke administration of HOE642 enhanced oligodendrogenesis and decreased apoptosis.

A. Quantitative analysis of $NG2^+Olig2^+$, $Ki67^+Olig2^+$, and $Caspase3^+Olig2^+$ cells in the CL and IL hemispheres of CC from the same cohort of mice in **Figure 3**, at 3 d post-stroke. N = 6-8. **B.** Quantitative analysis of $NG2^+Olig2^+$, $Ki67^+Olig2^+$, and $Caspase3^+Olig2^+$ cells in the CL and IL hemispheres of CC from the same cohort of mice in **Figure 3**, at 7 d post-stroke. N = 5. Data are mean ± SEM. * $p < 0.05$, ** $p < 0.01$, *** $p < 0.001$, **** $p < 0.0001$.

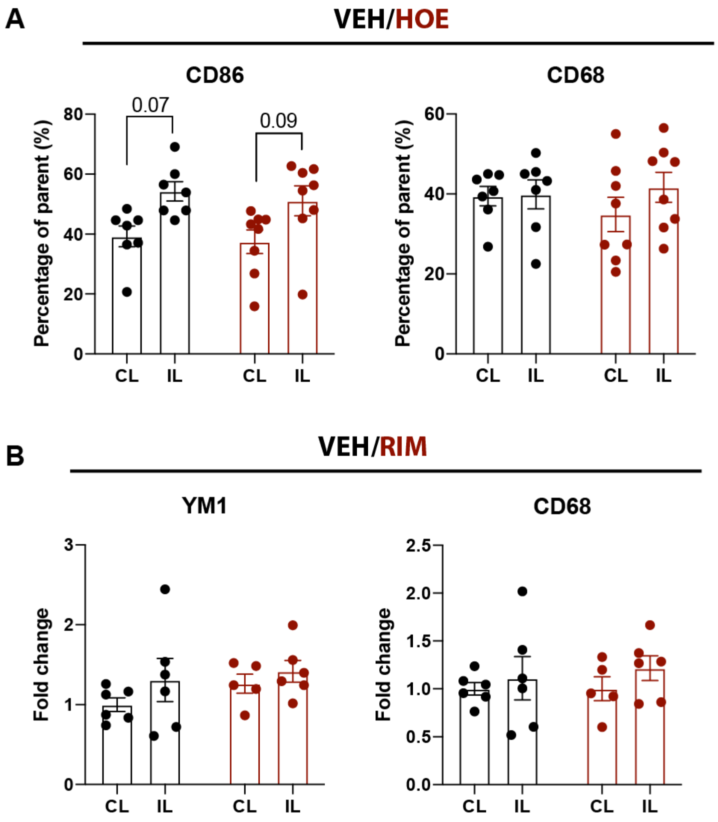

Figure S4. Additional profiling markers of microglia in HOE- or RIM-treated brains.

A. Quantitative analysis of inflammatory profiling markers CD86 and CD68 within the parent $CD11b^{+}CD45^{+}$ microglia/macrophages in the Veh- and HOE-treated mice at 3 d post-stroke. **B.** Quantitative analysis of anti-inflammatory markers Ym1 and CD68 within the parent $CD11b^{+}CD45^{+}$ microglia/macrophages in the Veh- and RIM-treated mice at 3 d post-stroke. Data from the same cohort of mice in **Figure 6**.

References:

1. Song, S.; Wang, S.; Pigott, V. M.; Jiang, T.; Foley, L. M.; Mishra, A.; Nayak, R.; Zhu, W.; Begum, G.; Shi, Y.; Carney, K. E.; Hitchens, T. K.; Shull, G. E.; Sun, D., Selective role of Na(+) /H(+) exchanger in Cx3cr1(+) microglial activation, white matter demyelination, and post-stroke function recovery. *Glia* **2018,** *66* (11), 2279-2298.
2. Song, S.; Yu, L.; Hasan, M. N.; Paruchuri, S. S.; Mullett, S. J.; Sullivan, M. L. G.; Fiesler, V. M.; Young, C. B.; Stolz, D. B.; Wendell, S. G.; Sun, D., Elevated microglial oxidative phosphorylation and phagocytosis stimulate post-stroke brain remodeling and cognitive function recovery in mice. *Commun Biol* **2022,** *5* (1), 35.
3. Swanson, R. A.; Morton, M. T.; Tsao-Wu, G.; Savalos, R. A.; Davidson, C.; Sharp, F. R., A semiautomated method for measuring brain infarct volume. *J Cereb Blood Flow Metab* **1990,** *10* (2), 290-3.
4. Huang, H.; Bhuiyan, M. I. H.; Jiang, T.; Song, S.; Shankar, S.; Taheri, T.; Li, E.; Schreppel, P.; Hintersteininger, M.; Yang, S. S.; Lin, S. H.; Molyneaux, B. J.; Zhang, Z.; Erker, T.; Sun, D., A Novel Na(+)-K(+)-Cl(-) Cotransporter 1 Inhibitor STS66* Reduces Brain Damage in Mice After Ischemic Stroke. *Stroke* **2019,** *50* (4), 1021-1025.
5. Kraeuter, A. K.; Guest, P. C.; Sarnyai, Z., The Y-Maze for Assessment of Spatial Working and Reference Memory in Mice. *Methods Mol Biol* **2019,** *1916*, 105-111.
